# Podocyte Depletion in Thin GBM and Alport Syndrome

**DOI:** 10.1371/journal.pone.0155255

**Published:** 2016-05-18

**Authors:** Larysa Wickman, Jeffrey B. Hodgin, Su Q. Wang, Farsad Afshinnia, David Kershaw, Roger C. Wiggins

**Affiliations:** 1 Department of Pediatrics and Communicable Diseases, University of Michigan, Ann Arbor, Michigan, United States of America; 2 Department of Pathology, University of Michigan, Ann Arbor, Michigan, United States of America; 3 Department of Internal Medicine, University of Michigan, Ann Arbor, Michigan, United States of America; UCL Institute of Child Health, UNITED KINGDOM

## Abstract

The proximate genetic cause of both Thin GBM and Alport Syndrome (AS) is abnormal α3, 4 and 5 collagen IV chains resulting in abnormal glomerular basement membrane (GBM) structure/function. We previously reported that podocyte detachment rate measured in urine is increased in AS, suggesting that podocyte depletion could play a role in causing progressive loss of kidney function. To test this hypothesis podometric parameters were measured in 26 kidney biopsies from 21 patients aged 2–17 years with a clinic-pathologic diagnosis including both classic Alport Syndrome with thin and thick GBM segments and lamellated lamina densa [n = 15] and Thin GBM cases [n = 6]. Protocol biopsies from deceased donor kidneys were used as age-matched controls. Podocyte depletion was present in AS biopsies prior to detectable histologic abnormalities. No abnormality was detected by light microscopy at <30% podocyte depletion, minor pathologic changes (mesangial expansion and adhesions to Bowman’s capsule) were present at 30–50% podocyte depletion, and FSGS was progressively present above 50% podocyte depletion. eGFR did not change measurably until >70% podocyte depletion. Low level proteinuria was an early event at about 25% podocyte depletion and increased in proportion to podocyte depletion. These quantitative data parallel those from model systems where podocyte depletion is the causative event. This result supports a hypothesis that in AS podocyte adherence to the GBM is defective resulting in accelerated podocyte detachment causing progressive podocyte depletion leading to FSGS-like pathologic changes and eventual End Stage Kidney Disease. Early intervention to reduce podocyte depletion is projected to prolong kidney survival in AS.

## Introduction

Genetic variants of the *COL4A3*, *COL4A4*, and *COL4A5* genes encoding α3, α4 and α5 type IV collagen polypeptide chains cause structural alterations of the glomerular basement membrane (GBM) that comprise both Alport Syndrome and Thin GBM Disease [1,2]. In this report these conditions are collectively designated as the Alport Syndrome Complex (ASC) to emphasize that they represent a spectrum of genetic and clinical phenotypes. ASC clinical phenotypes range from rapid progression to end stage kidney disease (ESKD) in early childhood to microscopic hematuria with stable normal kidney function lasting for >80 years [2,3]. During late glomerular development podocytes express, assemble and secrete α3, α4 and α5 collagen IV heterotrimers that become inserted into the GBM to replace α1, α1 and α2 collagen IV heterotrimers used to format the GBM during early glomerular development [4]. Mature GBM is a 300–400 nm thick (330±50 in males and 305±45 nm in females) highly ordered trilaminate α3, α4 and α5 type IV collagen-containing GBM which is resistant to proteolysis [1,5,6].

More than 1,000 genetic variants so far recognized in the large α3, α4 and α5 collagen IV genes are manifest by alterations of GBM structure identified in kidney biopsies by electron microscopy [2]. In more severe clinical phenotypes typical of major mutations of the *COL4A5* gene (classical “Alport Syndrome”) assembly of α3, α4 and α5 collagen IV heterotrimers within podocytes and their export into the GBM does not occur [1–3]. The resultant defective GBM has characteristic alternating thinning and thickening with splitting (lamellation) of the lamina densa accompanied by patchy podocyte foot process effacement and focal and segmental glomerulosclerosis associated with global glomerulosclerosis, interstitial fibrosis and culminating in ESKD. Since the *COL4A5* gene is resident on the X chromosome, classical AS affects males severely while females with random X inactivation in their podocyte population have a somewhat milder phenotype now recognized to cause end stage kidney disease (ESKD) in older age (15% by 60 years) [2,6].

Abnormal uniform thinning of the GBM (<250 nm thick) can be present at early stages of what will subsequently develop into the classical Alport GBM (alternating thick and thin segments and lamellation of the lamina densa) [7,8]. However, uniformly thinner than normal GBM is also a manifestation of less severe *COL4A5* genetic variants as well as variants of the *COL4A3* and *COL4A4* genes which are resident on chromosome 2 so their phenotypes are inherited in an autosomal recessive manner [2,7,8]. While classical Alport Syndrome is rare (3% of children with ESKD), thin GBM variants are reported to affect up to 1% of the population where they are increasingly recognized as contributing to renal failure occurring later in life, and in one series are reported to be four times as frequent a contributor to CKD and ESKD compared to classical AS [2]. The increasing recognition that FSGS in the presence or absence of thin GBM is also associated with *COL4A3* and *COL4A4* variants and may constitute a significant genetic cause of FSGS emphasizes the broad phenotype of the ASC family of diseases [9–13].

Glomerular diseases can be considered as a spectrum of podocytopathies [14–17]. In model systems progressive podocyte depletion *per se* has been proven to cause progressive glomerulosclerosis and progression to ESKD [18–19]. Genetic causes of FSGS and progressive glomerulosclerosis are caused by mutations in genes expressed by podocytes (e.g. *NPHS2* coding for podocin) [20]. The above observations both independently demonstrate that progressive glomerulosclerosis is caused by podocyte dysfunction and depletion. Furthermore, in observational studies of human glomerular diseases (diabetic glomerulosclerosis, IgA nephropathy, hypertensive glomerulosclerosis and transplant glomerulopathy) the progression process is also associated with podocyte depletion [21–27].

In model systems progression is accompanied by glomerular destabilization manifest by an increased rate of podocyte detachment into the glomerular filtrate which can be non-invasively monitored through urine pellet podocyte mRNAs [28–30]. All progressive glomerular diseases in man, including Alport Syndrome, are also associated with an increased rate of podocyte detachment as identified by the urine podocin mRNA:creatinine ratio [31]. This result therefore raised the question as to whether progression in ASC could be the result of progressive podocyte depletion from glomeruli. To address this question we measured podometric parameters in biopsies representing the spectrum of GBM abnormalities observed in ASC.

## Materials and Methods

Archival kidney biopsies obtained for routine clinical diagnostic purposes with a diagnosis of AS from subjects ≤18 years old over a 10 year period were used for analysis. Since podometric values are age-dependent the data used are restricted to the age-group <18 years so that they can be compared with a previously reported age-matched control group <18 years obtained from deceased kidney donors at time of transplantation [32]. The University of Michigan Institutional Review Board (HUM00025707, HUM00055525 and HUM00083116) waived the need for informed consent for use of these archival samples.

### Podometric methodology

The principles of the methodology used are outlined below. The referenced articles provide methodological details for sources of reagents, details of imaging and validation [27,32,33]. **(i) Estimation of podocyte numerical nuclear density [33].** Briefly, TLE4 immunofluorescence is used to identify and delineate podocyte nuclei in a regular paraffin-embedded formalin-fixed kidney biopsy section of known thickness (T) containing at least 8 glomerular profiles. Every glomerular profile in the biopsy is imaged in order using a calibrated microscope. Software is used to count nuclear profiles in each glomerulus and to measure the observed nuclear mean caliper diameter (d). A simple count of podocyte nuclear number per glomerular profile will overestimate true podocyte number by an amount that depends on the section thickness because tissue sectioning cuts podocyte nuclei such that each nucleus is represented in more than one section. A correction factor (CF) can be used to account for this problem and thereby estimate the true number of podocyte nuclei in a given volume of glomerular tuft. The CF = 1/(D/T+1) where T is the section thickness (that is known or can be measured) and D is the true mean caliper diameter of podocyte nuclei in the section (describing both nuclear size and shape). A quadratic equation (D = [(d-T) + sqrt ((d-T)^2 + 4kdT)]/2k) is used to estimate a value for D where d is the measured mean caliper diameter from the biopsy, T is the section thickness, and k is the shape value (which for podocyte nuclei = 0.72).

A downloadable spreadsheet in which the quadratic equation is embedded is provided (31) from which (by entering measured values for T, d, k, the number of nuclear profiles counted and the glomerular tuft area in which the podocyte nuclei were counted) values for true podocyte nuclear mean caliper diameter (D), numerical podocyte nuclear density and its reciprocal (average glomerular tuft volume per podocyte) are automatically generated. **(ii) Glomerular volume and podocyte volume**. Following imaging of the above TLE4 immuno-stained sections the coverslip is removed and the section re-stained using Glepp1 immuno-peroxidase, counterstained with hematoxylin, and then re-cover-slipped [27,32]. Each glomerulus is then re-imaged in the same order using the calibrated microscope. Using software the area of each glomerular tuft is measured and the % of that area that is peroxidase-stained (containing podocytes representing the proportion of the glomerular area occupied by podocyte cytoplasm) is estimated by software masking of the histochemical peroxidase product. The average glomerular tuft area measured in at least 8 consecutive glomerular profiles is used to estimate average glomerular volume according to Weibel and Gomez [34,35]. The estimated glomerular tuft volume occupied by podocytes is calculated by multiplying the GV by the Glepp1 positive % area. **(iii) Estimation of average podocyte number per tuft and average podocyte cell volume.** By knowing the average podocyte numerical nuclear density (number per volume) and the average glomerular tuft volume the average podocyte nuclear number per tuft can be estimated. By knowing the total podocyte volume (GV x Glepp1% area) and the average podocyte number per tuft a value the average volume of each podocyte can be estimated. **Estimate of % podocyte depletion:** The average podocyte number per tuft in each biopsy is compared as a % with the average podocyte number per tuft in the age and sex-matched control group. The concept of % podocyte depletion is used to compare AS data with similar data derived from rat model systems.

### Statistical Analysis

For descriptive purpose, mean and standard deviation (SD) was used in normally distributed continuous variables. An independent t-test was used to compare values for the control group with all ACS. Analysis of variance was used to compare the means of the ACS subgroups with that of the control group. Linear regression model was applied to quantify the relationship between the continuous variables. Spearman’s non-parametric rank coefficient was used for histologic scoring. Best fit curve estimation, including quadratic model was applied to demonstrate the non-linear associations between two continuous variables. Analyses were performed using SPSS version 21 (Armonk, New York).

## Results

**Fig 1:**
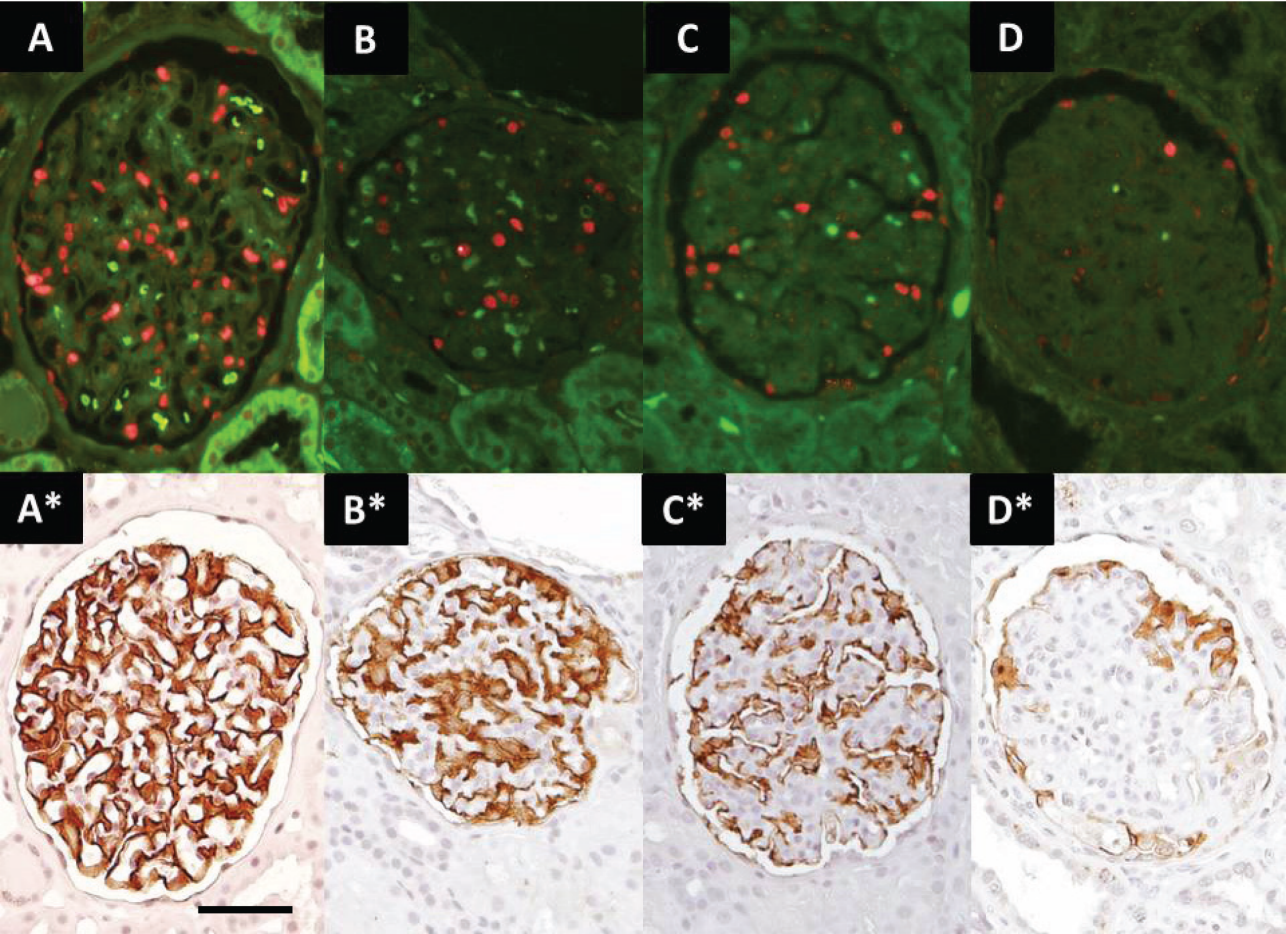
Identification of podocyte nuclei and cells in ASC biopsies. Upper panels show podocyte nuclei (red) identified by TLE4 immunofluoresce. Lower panels show Glepp1 immunoperoxidase (brown) in the same sections. Panels A and A* shows a normal glomerulus. Panels B to D and B* to D* show glomeruli with progressively reduced numbers of podocytes and Glepp1 peroxidase positive area (as a % of the glomerular area) representing progressive glomerular injury associated with AS. Parietal podocytes are excluded from analysis by delineating the area of interest using software so that the podocyte nuclear count and Glepp1 area quantitation is for the tuft area only. Magnification is the same for all panels where the bar shown represents 50um.

**Fig 2:**
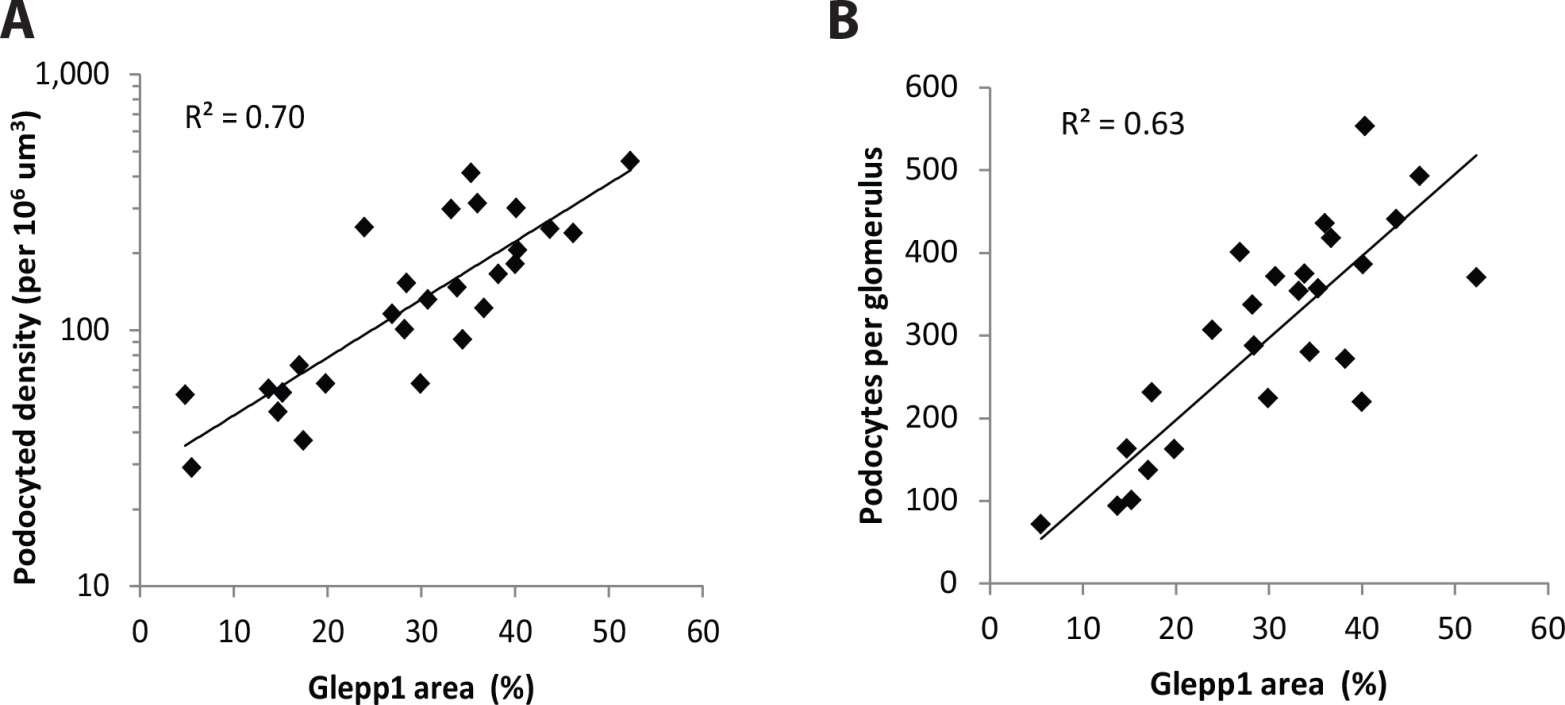
Comparison of two methods for measuring podocyte density. **Panel A**. Log podocyte density plotted versus Glepp1 area (%). The two methods correlate with R^2^ = 0.70. **Panel B**. Podocyte density x glomerular volume gives the podocyte number per tuft. This value also correlates well with Glepp1 area (R^2^ = 0.63). Bland-Altman comparison of podocyte density and podocyte number per glomerulus shows a mean difference of 106 (95% confidence interval of 115 to 327), and the Pitman’s test of difference in variance reveals r = 0.072, p = 0.728 indicating comparable results.

Biopsies from 21 patients with ASC were evaluated (Table 1). Podocyte nuclear density and cell area (Glepp1) density were measured in the same 3 um thick formalin-fixed paraffin-embedded histologic section as illustrated in Fig 1 (methods). Podocyte density estimated as podocyte nuclear numerical number per glomerular volume or Glepp1 positive area as a % of tuft area gave comparable results (Fig 2).

**Table 1:** ASC cohort characteristics at time of biopsy and treatment/outcome data. The deceased donor controls (n = 17) came from age 4–17 years deceased kidney donors with females/males = 12/5 similar to the ASC patient set (14/7).

Patient characteristics	Number	Qualifiers
ASC patients	21	
**Clinical characteristics**		
Age at time of biopsy	9.2±4.8	Range 2–17 years
Females/Males	14/7	
**Electron microscopic appearance**		
Classical Alport Syndrome pathology	16	20 biopsies
Thin GBM	5	6 biopsies
**Clinical Outcome**		
Did not progress to ESKD	14	14 biopsies
Progressed to ESKD requiring RRT	7	12 biopsies
**Treatment**		
ACE inhibitor	16	

**Comparison of ASC and control cohorts:** Table 2 (upper data sets) shows age-matched controls compared to the ASC cohort. Glomerular volume was not different between any groups or sub-groups. However podocyte number per glomerulus, podocyte nuclear density and Glepp1 positive cell area density were all significantly decreased in the ASC cohort.

**Table 2:** Clinical groupings showing podocyte depletion in ASC. Clinical parameters are shown to the left side and podometric parameters to the right side of the Table. Since age impacts podometric parameters the control group (n = 17 biopsies from deceased kidney donors aged 4–17 years) is appropriate for the ASC group (n = 26 biopsies from 21 patients aged 2–17 years). For cross-sectional analysis where more than one biopsy data set is available from one patient a single averaged value is used. Clinical parameters shown are from the time point at which the biopsy was performed. eGFR and urine protein:creatinine ratio (Urine PCR) data are not available (NA) for the control group. For each data set the mean (above in bold) and 1SD (below) are shown. Statistical comparisons of each data set compared to control using t-tests are shown by superscripted asterisks, while comparisons between sub-groups using analysis of variance are shown by asterisks in the area between the two sub-groups being compared. (* = P<0.05, ** = P <0.01). **Group Comparison:** All ASC subjects (n = 21) compared with control (n = 17) shows that glomerular volume was not different. However, podocyte number per glomerular tuft, podocyte nuclear density and podocyte cell (Glepp1 area density) were all decreased in ASC biopsies compared to control. Mean podocyte cell volume and mean podocyte nuclear diameter were not statistically significantly increased in ASC glomeruli. **GBM appearance:** Alport GBM is defined as typical alternating thin and thick GBM with lamellated lamina densa. Both the Thin GBM group (n = 5) and the Alport GBM group (n = 16) had significantly reduced podocyte number per tuft and Glepp1 area density compared to controls. The Thin GBM and Alport GBM groups were not significantly different from each other by any parameter (probably because one patient in the Thin GBM group progressed to ESKD in association with podocyte depletion). **Histologic appearance:** Biopsies containing only normal-appearing glomeruli without FSGS contained fewer podocytes and lower podocyte cell (Glepp1) area density than control. Biopsies with FSGS and/or global glomerulosclerosis (GGS) had significantly fewer podocytes (podocyte number per glomerulus, density or podocyte area density) than both control and ASC biopsies with normal appearing glomeruli. “**Non-progressors” vs Progressors**: Biopsies from people who did not progress (“Non-progressors”) as judged by retaining eGFR within the normal range by follow-up of 3 or more years (mean follow-up 7.4±3.9 years, n = 8) had significant podocyte depletion (reduced number of podocyte per glomerulus and reduced podocyte area [Glepp1 area density] compared to control. Biopsies from people who subsequently progressed to ESKD requiring renal replacement therapy (n = 7 patients) had significantly increased podocyte depletion at time of biopsy (reduced podocyte number per glomerulus, podocyte nuclear density and Glepp1 area density and larger podocytes) than those that did not progress.

		Age	eGFR	UrinePCR	Tufts/biopsy	FSGS	GlomVol	Pod NuclDiameter	Pod NuclNumber	Pod NuclDensity	Glepp AreaDensity	Av PodVolume
	n	yrs	ml/min	g/g	n	%	x10^6^ um^3^	um	n	per 10^6^ um^3^	%	um^3^
**Biopsy groups**												
**Control group**	**17**	**11.6**	**NA**	**NA**	**21.7**	**NA**	**1.9**	**6.8**	**511**	**309**	**44.0**	**1,610**
1SD		4.7			9.2		1.0	0.6	153	117	**4.8**	**560**
**All ASC biopsies**	**21**	**9.6**	**109**	**4.2**	**22.9**	**12.4**	**2.0**	**7.1**	316**	203**	31.8**	**2,032**
1SD		4.7	48	10.0	7.9	20.4	1.0	0.8	125	130	11.2	965
**GBM appearance**												
**Thin GBM**	**5**	**10.6**	**117**	**0.9**	**23.3**	**8.0**	**1.9**	**6.7**	359*	**209**	32.4**	**1,823**
1SD		3.2	25	1.1	6.0	11.1	0.7	0.6	107	91	9.3	847
		NS	NS	NS	NS	NS	NS	NS	NS	NS	NS	NS
**Alport GBM**	**16**	**9.2**	**107**	**5.3**	**22.8**	**13.8**	**2.0**	**7.2**	302**	202*	31.5**	**2,098**
1SD		5.1	54	11.3	8.6	22.8	1.1	0.8	131	143	12.0	1,016
**Histologic appearance**												
**Normal**	**11**	**8.6**	**129**	**0.7**	**23.5**	**0.2**	**1.8**	**7.0**	405*	**265**	39.6*	**1,713**
1SD		4.9	42	0.9	8.4	0.6	0.8	0.5	76	106	5.8	659
		NS	*	NS	NS	**	NS	NS	**	*	**	NS
**FSGS/GGS**	**10**	**10.6**	**84**	**8.1**	**22.3**	**24.6**	**2.3**	**7.1**	219**	137**	23.1**	2,384*
1SD		5.0	45	13.8	7.8	23.4	1.2	1.1	91	125	9.0	1,151
**Non-Progressors vs Progressors**												
**Non-progressors**	**8**	**8.8**	**111**	**0.4**	**19.9**	**4.3**	**1.7**	**6.8**	388*	**274**	38.5*	**1,625**
1SD		3.8	20	0.2	9	8.4	0.9	0.4	100	114	8.1	744
		NS	NS	NS	NS	**	NS	NS	**	*	**	*
**Progressors**	**7**	**10.2**	**76**	**11.2**	**22.6**	**26.7**	**2.4**	**7.1**	189**	119**	14.8**	2,558*
1SD		5.3	53	15.7	8.0	27.7	1.2	0.4	88	142	7.5	1,253

**Alport (thin/thick/lamellated) GBM versus Thin GBM biopsies:** The Alport sub-group and the Thin GBM sub-group both had significantly reduced podocyte parameters compared to control (Table 2). Although the Alport sub-group had greater numerical podocyte reduction than the Thin GBM sub-group there was no statistical difference between them. This reflects the fact that in the cohort studied one patient with Thin GBM progressed to ESKD associated with significant podocyte depletion.

**Normal histology versus biopsies with focal segmental or global glomerulosclerosis**: Biopsies with focal segmental or global glomerulosclerosis had markedly reduced podocyte parameters compared to control (Table 2). However, biopsies that had no detectable abnormality by light microscopy also had significant reduction in podocyte number per glomerulus and Glepp1 area density compared to controls. The “normal biopsy” sub-group was also significantly different from the “glomerulosclerosis” sub-group which had a greater level of podocyte depletion.

**“Non-progressors” versus “Progressor” groups**: “Non-progressors” defined as those who showed no significant reduction in renal function over at least a three year period of observation (mean 7.6±3.4 years) had reduced podocyte number per glomerulus and Glepp1 area density compared to controls (Table 2). Biopsies performed on those who would subsequently progress to ESKD requiring renal replacement therapy (defined as “Progressors”) had a greater level of podocyte depletion as measured by all podometric parameters as well as increased podocyte cell volume. At the time of biopsy the “progressor” group had significantly lower eGFR than the “non-progressor” group although the average eGFR was in the normal range (83±42ml/min) associated with higher level proteinuria (Urine PCR 9.6±18.2) and a higher level FSGS (41.8±33% of glomeruli).

**Fig 3:**
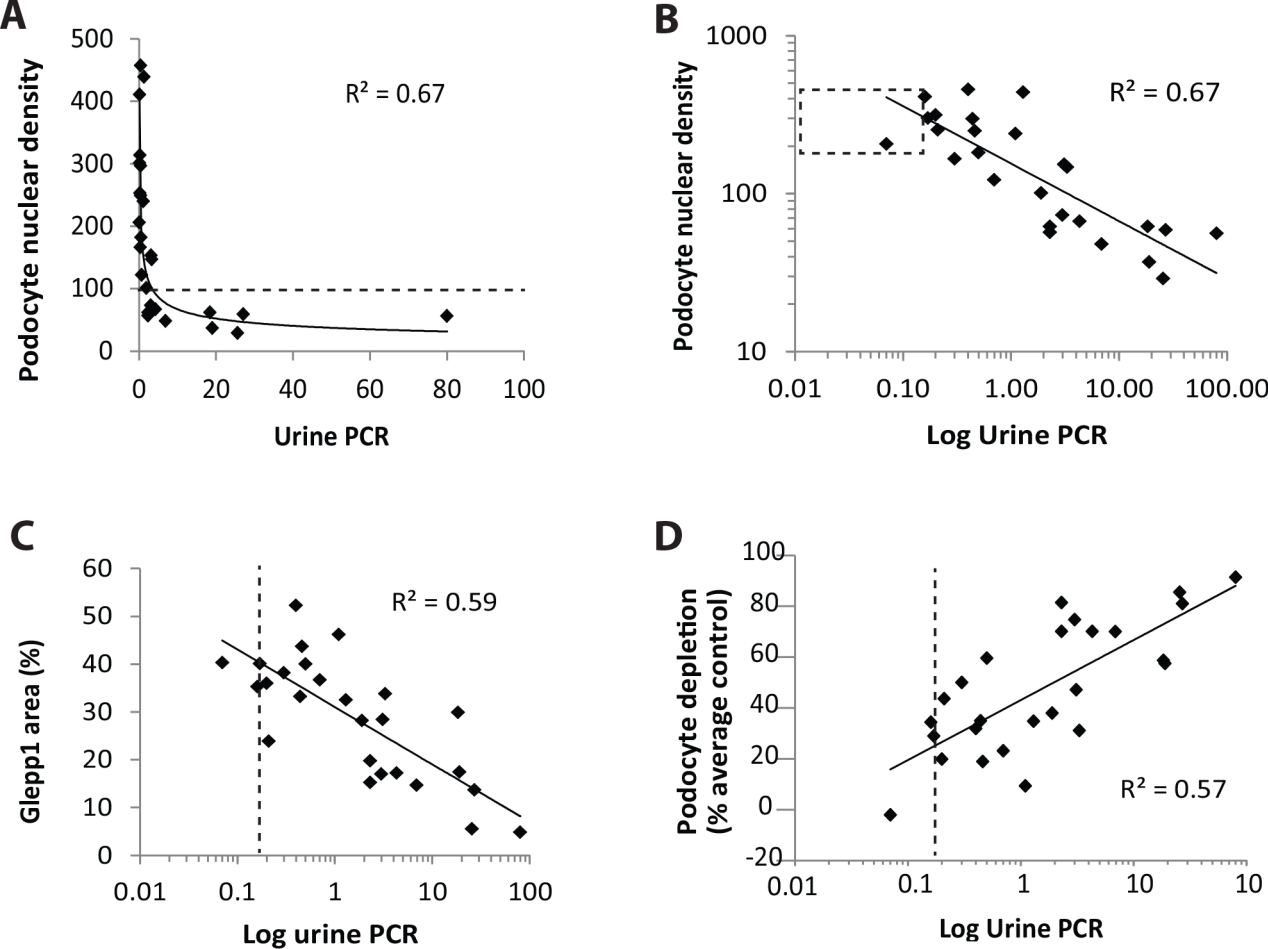
Proteinuria increases in proportion to podocyte number per glomerular tuft and podocyte density decrease. **Panel A:** Urine PCR shown on a linear scale in relation to podocyte density demonstrating that as podocyte density decreases below approximately 100 per 10^6^ um^3^ the level of proteinuria exponentially increases. **Panel B:** The same data as shown in Panel A except that proteinuria and podocyte density are shown on a log scale. The dotted box represents the mean±1SD for podocyte density and the upper limit of normal urine PCR at 0.18. Note that the log scale shows that proteinuria increases in proportion to reduction in podocyte density over a wide range. **Panel C:** Glepp1% area is an alternative way of measuring podocyte density also shows an exponential (log) relationship with proteinuria. **Panel D:** % podocyte depletion (calculated as a % of the normal age-matched mean level for each sample) shown in relation to log urine PCR demonstrating that at the upper limit of normal urine PCR (0.18) occurs when approximately 20% of podocytes have been lost. By the time the urine PCR has reached a value of 1 about 40% of podocytes have been lost. These data illustrate that low level proteinuria is a moderately early marker of podocyte depletion in ASC biopsies.

**Degree of proteinuria is related to the degree of podocyte depletion.** The relationship between proteinuria (as assessed by the urine protein:creatinine ratio) and podocyte depletion is shown in Fig 3. In Fig 3A proteinuria is shown as a linear plot in relation to podocyte density to indicate that higher level proteinuria occurs when the podocyte density falls below 100 per 10^6^ um^3^. Fig 3B shows the same plot except on a log scale to indicate that reduction in podocyte density lower than 1SD below the mean (shown by the dashed line box) is associated with proportionally increased proteinuria over a wide range. Fig 3C shows that proteinuria also increases in association with reduced podocyte density as measured by the Glepp1 area % as an independent measure of podocyte density. Fig 3D shows the relationship between % podocyte depletion and log urine PCR with the upper limit of normal urine PCR indicated by the vertical dotted line to illustrate that proteinuria level becomes abnormal after about 25% podocyte depletion. Degree of proteinuria is therefore directly related to the amount of podocyte depletion, and is a moderately sensitive measure of degree of podocyte depletion from glomeruli.

**Fig 4:**
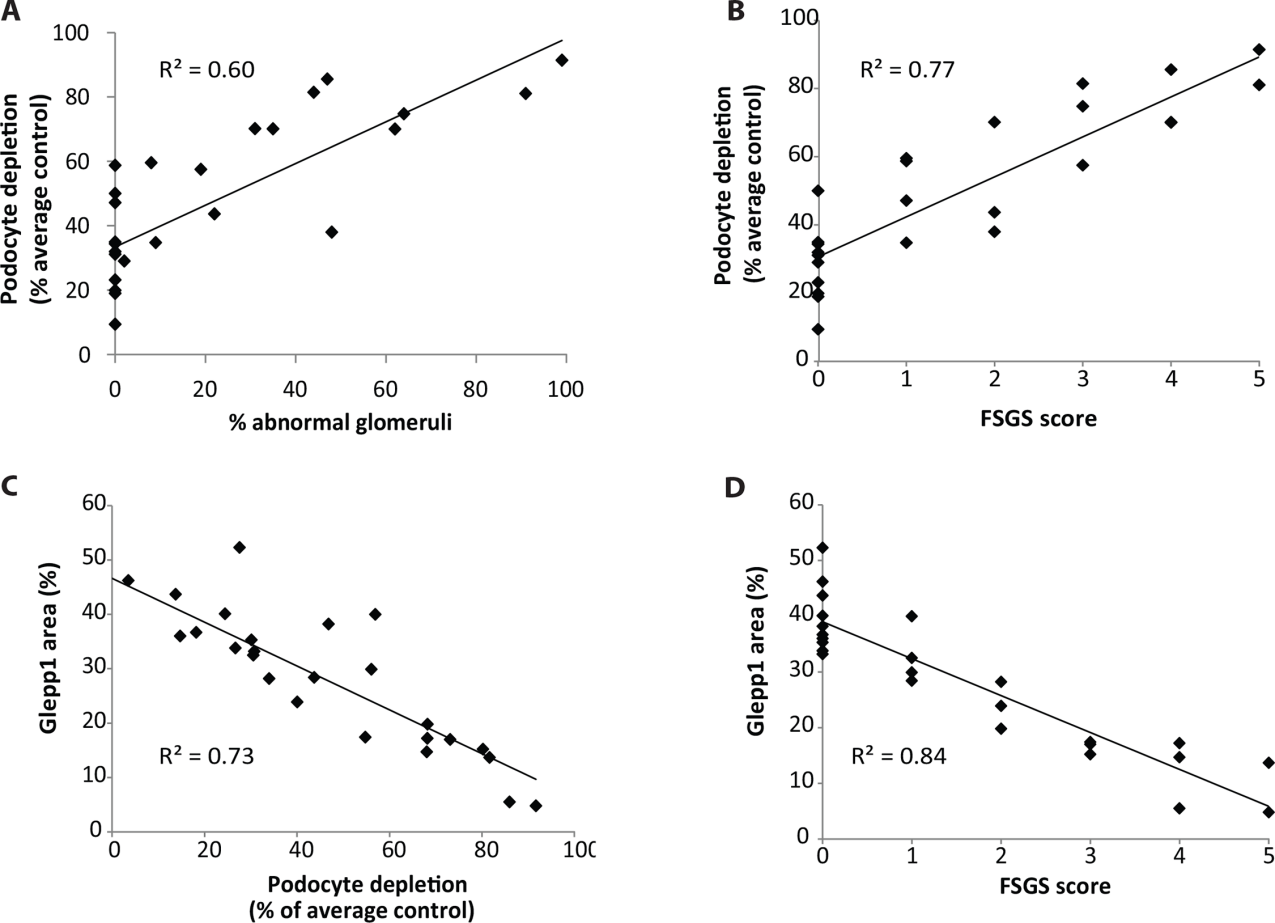
Histologic parameters increase in proportion to podocyte depletion. **Panel A:** The proportion of histologically detectable abnormal glomeruli present in a biopsy increases linearly in relation to the decrease in podocyte number per glomerulus after approximately 30% of podocytes have been lost. **Panel B:** Biopsies are scored as within normal limits (score 0), mesangial expansion only (score 1), or adhesions to Bowman’s capsule (score 2) or FSGS affecting <10% of glomeruli (score 3) or FSGS affecting >10% of glomeruli (score 4) or widespread glomerulosclerosis and interstitial fibrosis (score 5) increases. As the degree of podocyte depletion increases above 30% the histologic injury score increases. Note that no glomerular changes are present below 30% depletion, minor glomerular changes are associated with <50% depletion, major glomerular changes (FSGS and global glomerulosclerosis) are present above 50% podocyte depletion. **Panel C and D:** The Glepp1 area % as an alternative method of measuring podocyte density is highly correlated with the % podocyte depletion (R^2^ = 0.73) and FSGS scoring (R^2^ = 0.84). Spearman correlation coefficients are used in all panels.

**Histologic parameters in relation to degree of podocyte depletion.** Biopsies were scored blinded by JH using two scoring systems. In the first the proportion of glomeruli demonstrating any abnormality including mesangial expansion, adhesions to Bowman’s capsule, FSGS and global GS were added up and expressed as % abnormal glomeruli present in the biopsy. In the second the “FSGS score” was measured by determining whether biopsies contained normal glomeruli only (score 0), mesangial expansion only (score 1), adhesions to Bowman’s capsule (score 2), <10% of glomeruli with FSGS (score 3), >10% of glomeruli with FSGS (score 4) and widespread glomerulosclerosis and interstitial fibrosis (score 5). In Fig 4A the % abnormal glomeruli is plotted against the % podocyte depletion (compared to the age-matched control value). There is a linear relationship between reduction in podocyte number and the % of abnormal glomeruli in the biopsy (R^2^ = 0.57). Podocyte depletion greater than 30% is associated with an increasing proportion of abnormal glomeruli. Fig 4B shows % podocyte depletion in relation to the FSGS score. Again no abnormality is observed below 30% podocyte depletion. FSGS is present at >50% podocyte depletion. Fig 4C and 4D show similar data using Glepp1 area as the measure of podocyte depletion. The correlations are particularly high because the Glepp1 area % quantitates the same parameter as is being scored by the pathologist (i.e. the proportion of the glomerular tuft that is abnormal). Collectively these data demonstrate that no abnormality is detected histologically below 30% podocyte depletion and that more than 50% podocyte depletion has to occur before FSGS is present in ASC biopsies. Histologic changes therefore occur in relation to podocyte depletion but are a relatively insensitive measure of degree of podocyte depletion.

**Fig 5:**
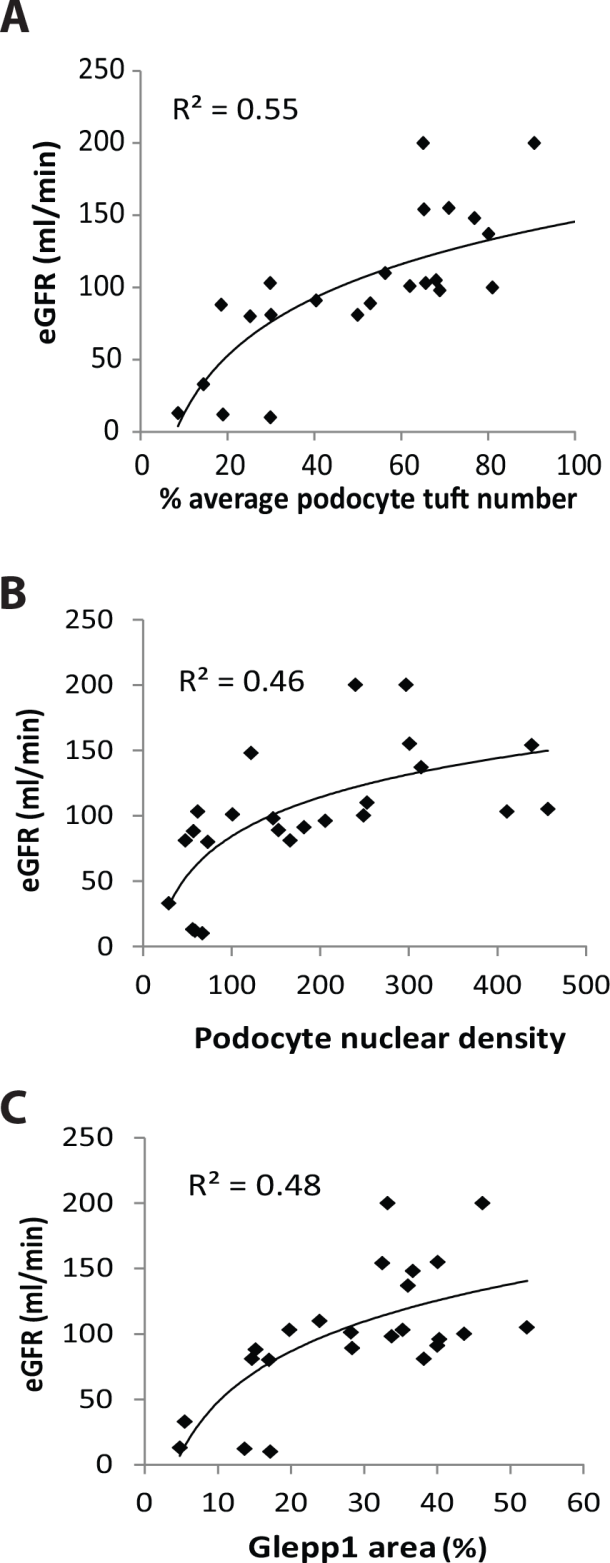
Reduced eGFR is a late marker of podocyte depletion. **Panel A**: Kidney function as measured by eGFR is detectably reduced only when podocyte depletion is reduced to the level of about 20% of normal (i.e. 80% depletion). **Panel B:** eGFR becomes measurably reduced when podocyte density falls below 100 per 10^6^ um^3^. **Panel C:** eGFR becomes measurably reduced when the Glepp1% area is reduced below about 15–20%. Two eGFR values of 380 and 400 ml/min from an infant aged 2 and 3 years with a serum creatinine of 0.1mg% and high level proteinuria at the time of biopsy were excluded from analysis because of the inherent inaccuracy of the eGFR estimate under those conditions. These data collectively show that reduction in eGFR below the normal range (60ml/min) is a late marker of podocyte depletion in ASC biopsies. Logarithmic equations using best fit curve estimation are used in all panels.

**Estimated glomerular filtration rate (eGFR) in relation to degree of podocyte depletion.** The relationship between eGFR and podocyte parameters is shown in Fig 5. eGFR does not become measurably abnormal until approximately 70% of podocytes have been lost and the podocyte density is well below 100 per 10^6^ um^3^. Measurably reduced eGFR is therefore a late marker of podocyte depletion.

## Discussion

The focus on podocyte depletion in ASC was precipitated by our finding that the rate of podocyte detachment as measured by urine podocyte mRNA markers was increased in Alport Syndrome urine [31]. We now show that podocyte depletion as measured by podocyte number per glomerulus, % podocyte depletion compared to an age-matched control group, or Glepp1 positive % of tuft area [podocyte cellular density], all demonstrate that podocyte depletion occurs in association with ASC. This result is not unexpected in glomeruli that have established focal segmental or global glomerulosclerosis. However podocyte depletion was already present in apparently normal-appearing glomeruli from biopsies with both typical Alport GBM (alternating thin and thick segments with lamellated lamina densa) and uniformly Thin GBM. Depletion of about 30% of podocytes is required before detectable early histologic abnormalities (e.g. mesangial expansion) can be seen in these glomeruli, and FSGS is not apparent until >50% podocyte depletion has occurred. This result is similar to that previously documented in a transgenic rat model of podocyte depletion [19]. In this model, where podocytes can be selectively deleted at a predetermined time and by a predetermined amount, no histologic abnormality is detected until 20–30% of podocytes are deleted and FSGS is not present until >50% of podocytes are deleted. That (i) podocyte depletion is an early event preceding onset of glomerulosclerosis in ASC, (ii) the degree of glomerular abnormality is directly proportional to the degree of podocyte depletion in ASC, and (iii) this quantitative relationship is reproduced in a model of specific podocyte deletion where we know that podocyte depletion *per se* is the causative mechanism, provides strong support for the hypothesis that podocyte depletion is also a significant factor driving the progression process in ASC.

Compelling data supports the concept that progressive podocyte injury and depletion drives glomerular disease progression (glomerulosclerosis) in most, if not all, glomerular diseases. This conclusion stems from model systems (where specific podocyte depletion is shown to drive development of the FSGS phenotype), genetic variants in podocyte-specific genes (that cause an FSGS phenotype), and observational studies of kidney biopsies from diabetic glomerulosclerosis, IgA Nephropathy, hypertensive glomerulosclerosis and transplant glomerulopathy [14–27]. AS therefore represents another example of this paradigm. Podocyte injury and depletion is also a source of proteinuria that is well-known to be strongly associated with worse outcome in all progressive glomerular diseases [36]. The podcyte depletion hypothesis provides a plausible mechanistic explanation for the hyperfiltration hypothesis of Brenner and colleagues [37]. Kriz and Le Hir evaluate protein in the filtrate *per se* as unlikely to be a major progression mechanism [16]. Fibrotic processes in both the glomerular and interstitial compartments also contribute to the progression process and may play a special role in AS [38,39].

The inability of podocytes to synthesize and export normal α3, α4 and α5 collagen IV heterotrimers to maintain a normal trilaminate 300–400 nm wide GBM in ASC patients could potentially result in impaired podocyte adherence to GBM leading to accelerated podocyte detachment. This concept was previously suggested as a possible mechanism for the FSGS-like pathology found in Alport syndrome by Le Hir and Kriz [16,17]. Depending on the degree of impaired podocyte adherence and over time this mechanism could cause progressive podocyte depletion from glomerular tufts. These early events are associated with low level proteinuria [19]. When podocyte depletion exceeds about 30% of podocytes early pathologic changes appear (mesangial expansion and adhesions to Bowman’s capsule) and proteinuria increases. When podocyte depletion exceeds 50% FSGS-like lesions appear as glomeruli respond by patching leaky areas with matrix material that with further podocyte depletion causes major FSGS and global glomerulosclerosis with high level proteinuria, interstitial fibrosis, reduced renal function, and ultimately, progression to ESKD. Accumulating reports showing that *COL4A3* and *COL4A4* variants cause FSGS-like pathology in the presence or absence of detectable abnormalities of GBM structure would also be compatible with these data [9–13]. This concept opens up the possibility that podocyte adherence could be directly targeted as a means to prevent podocyte detachment and thereby delay or prevent progression to ESKD in AS.

Proteinuria is a marker of increased risk for progression in AS with the recommendation that treatment with ACE inhibition be initiated when the urine protein:creatinine ratio becomes increased above normal [40–42]. We project that on average podocyte number per tuft will already be decreased by about 25% by the time the urine PCR is above the normal range. Although the eGFR of a sub-group of patients (“non-progressors”) was not measurably reduced at time of biopsy even after an average follow-up time of 7.4±3.9 years, these patients already had significantly reduced podocyte parameters. As emphasized by recently reported glomerular aging data this degree of podocyte depletion would be expected to result in higher risk for premature age-associated ESKD occurring in later life [32]. Taken together these data suggest that the earlier ASC patients can be identified and the intervention to reduce their rate of podocyte detachment implemented the longer ESKD-free life can be expected. This approach is projected to impact not only classical AS but also less severe ASC phenotypes.

Measurable reduction in eGFR was not apparent until >70% of podocytes have been lost. Similar findings are reported in diabetic glomerulosclerosis, IgA nephropathy and Transplant glomerulopathy [21–27]. The concept that by the time eGFR is measurably decreased major podocyte depletion has already occurred emphasizes that eGFR is an insensitive approach for identifying risk for progression.

In the hDTR rat model of specific podocyte depletion the protective effect of angiotensin II blockade can entirely be accounted for by its effect on reducing the rate and amount of podocyte depletion from glomerular tufts [29]. Angiotensin II-dependent podocyte detachment occurs in association with increased filtration pressure and an altered podocyte phenotype (e.g. reduced nephrin expression and altered actin cytoskeleton) that together facilitate accelerated podocyte detachment from the GBM [43]. Progression to ESKD is also reported to be slowed by angiotensin II blockade in AS [40], compatible with a similar angiotensin II-dependent podocyte loss mechanism playing a role in driving progression in ASC.

As outlined in the Introduction classical AS represents a subset of the total ASC cohort that develops early ESKD. Typical X-linked AS affecting males with associated auditory and eye abnormalities and a family history of AS are frequently not biopsied and therefore are not proportionally represented in this and other biopsy series. Most biopsies in our cohort were performed for a combination of hematuria and at least low level proteinuria, rather than for hematuria alone. This would be expected to result in a greater proportion of biopsies having more significantly reduced podocyte parameters than would have been the case if biopsies were performed for isolated hematuria in the absence of proteinuria. However even in biopsies for hematuria alone it would be expected that the rate of podocyte detachment as measured by the urine podocyte detachment assay would be increased, and therefore could potentially provide a sensitive index of both the potential for progression and responsiveness to treatment.

Although the 21 patients with 26 biopsies used for this study was small the correlations and comparisons demonstrate important relationships describing degree of podocyte depletion in relation to clinical parameters. The wide range of podocyte depletion within the cohort allows the full extent of relationships of podocyte depletion to clinical parameters to be recognized. No genetic data were available for the cohort used so that the ASC diagnosis is based on typical ultrastructural electron microscopic findings in the appropriate clinical context. Side-by-side genetic, phenotypic and podometric analysis will be required to determine how these complementary perspectives can optimally be used for ASC clinical decision-making.

In summary this report supports a hypothesis that podocyte depletion might be what actually drives the progression process in ASC, as in other glomerular diseases. If so, measurement of podocyte density and number per tuft in kidney biopsies could potentially provide a quantitative readout describing risk for progression prior to the appearance of pathologic changes or detectable reduction in eGFR. In combination with the rate of podocyte detachment as measured by the urine assay this approach could potentially provide useful additional guidance for clinical decision-making.
